# Genome-wide analysis of bHLH transcription factor and involvement in the infection by yellow leaf curl virus in tomato (*Solanum lycopersicum*)

**DOI:** 10.1186/s12864-015-1249-2

**Published:** 2015-02-05

**Authors:** Jinyan Wang, Zhongze Hu, Tongmin Zhao, Yuwen Yang, Tianzi Chen, Mali Yang, Wengui Yu, Baolong Zhang

**Affiliations:** Provincial Key Laboratory of Agrobiology, Jiangsu Academy of Agricultural Sciences, Nanjing, China

**Keywords:** Genome-wide analysis, bHLH transcription factor, TYLCV, VIGS, Tomato

## Abstract

**Background:**

The basic helix-loop-helix (bHLH) proteins are a superfamily of transcription factors that can bind to specific DNA target sites. They have been well characterized in model plants such as *Arabidopsis* and rice and have been shown to be important regulatory components in many different biological processes. However, no systemic analysis of the bHLH transcription factor family has yet been reported in tomatoes. Tomato yellow leaf curl virus (TYLCV) threatens tomato production worldwide by causing leaf yellowing, leaf curling, plant stunting and flower abscission.

**Results:**

A total of 152 bHLH transcription factors were identified from the entire tomato genome. Phylogenetic analysis of bHLH domain sequences from Arabidopsis and tomato facilitated classification of these genes into 26 subfamilies. The evolutionary and possible functional relationships revealed during this analysis are supported by other criteria, including the chromosomal distribution of these genes, the conservation of motifs and exon/intron structural patterns, and the predicted DNA binding activities within subfamilies. Distribution mapping results showed bHLH genes were localized on the 12 tomato chromosomes. Among the 152 bHLH genes from the tomato genome, 96 bHLH genes were detected in the TYLCV-susceptible and resistant tomato breeding line before (0 dpi) and after TYLCV (357 dpi) infection. As anticipated, gene ontology (GO) analysis indicated that most bHLH genes are related to the regulation of macromolecule metabolic processes and gene expression. Only four bHLH genes were differentially expressed between 0 and 357 dpi. Virus-induced gene silencing (VIGS) of one bHLH genes *SlybHLH131* in resistant lines can lead to the cell death.

**Conclusion:**

In the present study, 152 bHLH transcription factor genes were identified. One of which bHLH genes, *SlybHLH131*, was found to be involved in the TYLCV infection through qRT-PCR expression analysis and VIGS validation. The isolation and identification of these bHLH transcription factors facilitated clarification of the molecular genetic basis for the genetic improvement of tomatoes and the development of functional gene resources for transgenic research. In addition, these findings may aid in uncovering an unexplored mechanism during the TYLCV infection in tomatoes.

**Electronic supplementary material:**

The online version of this article (doi:10.1186/s12864-015-1249-2) contains supplementary material, which is available to authorized users.

## Background

The basic/helix-loop-helix (bHLH) proteins have DNA-binding and dimerization capabilities. They are a superfamily of transcription factor (TFs) that have been found to have many different functions in essential physiological and developmental process in animals and plants [1-3]. The bHLH domain contains approximately 60 amino acids with two functionally distinct regions, the basic region and the HLH regions [4]. The basic region was 15 amino acids long and typically included six basic residues. It was located at the N terminus of the domain and functions as a DNA binding motif [5]. The HLH region contains two amphipathic α helices separated by a loop region of variable length. The HLH region acts as a dimerization domain and allows the formation of homodimers or heterodimers [1,6]. Among all bHLH motifs, 19 amino acids have been found to be highly conserved in organisms ranging from yeast to mammals [7]. Outside of these conserved bHLH domains, the proteins exhibited considerable sequence divergence. Some bHLH proteins have been shown to bind to the sequences containing the core element known as the E box (5′-CANNTG-3′), with the most common form of G-box (5′-CACGTG-3′). The nucleotides flanking the core element may also have a role in binding specificity [5,8].

Based on the phylogenetic relationships, DNA-binding motifs, and functional properties, the bHLH TFs family has been divided into six main groups in metazoans [2,9,10]. In brief, Group A bHLH proteins can bind to the CAGCTG core sequences of E-boxes. Group B includes a large number of functionally proteins (Max, Myc, MITF, and USF) and bind to the G-box sequence CACGTG [11,12]. Group C contains an additional protein-protein interaction region (the PAS domain) and binds to ACGTG or GCGTG sequences. Group D proteins have the HLH region but lack the basic DNA binding domain [13]. Group E proteins have Pro or Gly residues within the basic region and can bind preferentially to a typical sequence, CACGNG [14]. Group F consists of the COE domain; they have diverse sequences compared with other groups and another domain for dimerization and DNA binding [2,14,15].

Only a small number of plant bHLH proteins have been characterized functionally, far fewer than in animals. In *Arabidopsis*, 162 bHLH-encoding genes which were divided into 21 subfamilies according to their phylogenetic relationships have been identified from the analysis of genome sequences [3,16]. A total of 167 and 230 bHLH TFs have been identified in the rice (*Oryza sativa*) and Chinese cabbage (*Brassica rapa*) genomes, respectively [17,18]. These have been divided into 22 and 24 subfamilies, respectively. Phylogenetic analysis showed that the plant bHLH proteins comprised 26 subfamilies, 20 of which were present in the common ancestors of extant mosses and vascular plants [19]. Most bHLH proteins identified so far have been functionally characterized in *Arabidopsis*, and their roles have been shown to include regulation of fruit dehiscence, anther and epidermal cell development, hormone signaling, and stress responses [20].

Tomato (*Solanum lycopersicum*) is an economically important vegetable worldwide. The annual global production of tomato in 2012 was more than 160 million tons including 50 million tons in China (http://faostat.fao.org/). The tomato genome has been sequenced and assembled by the International Tomato Genome Sequencing Project (http://solgenomics.net/organism/Solanum_lycopersicum/genome), because tomatoes are economically important and it is model species for the study of fruit ripening [21]. A high-quality genome sequence for domesticated tomato and more than 30,000 proteins have been obtained. Tomato yellow leaf curl virus (TYLCV) is the most widespread and currently ranks 3^rd^ among the most economically and scientifically most important plant viruses worldwide [22]. The symptoms of TYLCV infection in young plants include stunted growth, upward curling of leaf margins, marked reduction in leaf size, mottling and yellowing of young leaves, and flower abscission, leading to severe yield loss [23]. Currently five major loci resistant to TYLCV have been identified from different wild tomato relatives, *Ty-1*, *Ty-3* and *Ty-4* from *S.chilense*, *Ty-2* from *S.habrochaites*, and *Ty-5* from *S.peruvianum* [24-28]. Among them, *Ty-1* and *Ty-3* were found to be allelic and have been cloned. *Ty-1* and *Ty-3* were found to be allelic and have been cloned. They are RNA-dependent RNA polymerases (RDR) and may be involved in RNA silencing [29]. In addition, *Ty-2*, *Ty-4* and *Ty-5* have been mapped to chromosomes 11, 3, and 4 respectively, using molecular markers [26,30-32]. cDNA library comparisons of susceptible and resistant tomato lines before and after TYLCV infection showed approximately 70 genes that are preferentially expressed in a tomato line with a resistance introgressed from *S. habrochaites* [29]. Using whole transcriptome sequencing of the TYLCV-resistant tomato breeding line CLN2777A (R) and TYLCV-susceptible tomato breeding line TMXA48-4-0 (S), 209 and 809 genes were found to be differentially expressed in the R and S tomato lines, respectively [33].

In tomatoes, *LeFER*, a bHLH protein encoded by Solyc06g051550.2.1, SlybHLH083, was the first identified regulator of iron nutrition in plants. *LeFER* plays an important role in the Fe-deficiency response of tomatoes [34]. *Style2.1*, encoded by Solyc02g084880.2.1, SlybHLH031, is the major quantitative trait locus responsible for style length; this important floral attribute has been shown to be associated with the evolution of self-pollination and was cloned in cultivated tomatoes [35]. However, the tomato bHLH protein family has not been analyzed at a genome-wide level, and the phylogenetic relationship of this protein family remains poorly understood. In this study, a total of 152 *SlybHLH* genes were identified in the tomato genomic sequence and phylogenetic analyses were carried out to evaluate the relationships among these genes. Changes in global expression pattern of *SlybHLH* genes in R and S lines infected by TYLCV were analyzed to provide insight into the regulation of response to TYLCV. The expression of *SlybHLH* exhibited a variety of expression patterns, suggesting a novel layer of regulation for the response to TYLCV in tomato.

## Methods

### Database search for bHLH genes

The Pfam database (http://pfam.sanger.ac.uk/) [36] was used to screen the genome of tomato (*S. lycopersicum*; http://solgenomics.net/organism/Solanum_lycopersicum/genome) and potato (*S. tuberosum*; http://phytozome.jgi.doe.gov/). Proteins with helix-loop-helix DNA-binding domains (PF00010.21) were used to identify the putative bHLH proteins in tomato and potato using the hidden Markov model (HMM). The hmmsearch tool, with an expected value (e-value) cut-off of 1.0 was used to identify the proteins. These sequences were then verified using the SMART tool (http://smart.embl-heidelberg.de/) [37]. The *Arabidopsis thaliana* bHLH proteins were retrieved from the TAIR database (http://www.arabidopsis.org/) using a previous report [3].

### Phylogenetic analysis and identification of conserved motifs and gene structure

The complete amino acid sequences were screened against the Pfam database to identify the domains of bHLH transcription factors. MEGA6 software was used to construct neighbor-joining (NJ) distance trees using tomato bHLH protein domain sequences [38]. The bootstrap was set as 1,000 replicates, which provided information regarding their statistical reliability. Meanwhile, the NJ method of the PHYLIP software (version 3.6; http://evolution.genetics.washington.edu/phylip.html; [39]) was also used with bootstrap of 1000 replicates to create another phylogenetic tree to validate the results from the NJ method by MEGA 6 software. A phylogenetic tree of all the identified bHLH protein domains was also constructed. The identified bHLH domains were aligned using a ClustalX 2.0 program with default settings [40].

To identify the conserved motifs in tomato bHLH proteins, the Multiple Expectation-maximization for Motif Elicitation (MEME) program version 4.9.0 [41] was used with default parameters, except for the following parameters: (1) optimum motif width was set to ≥10 and ≤100; (2) the maximum number of motifs was set to identify ten motifs. MEME software (http://meme.sdsc.edu/meme/) was used to search for conserved motifs in the complete amino acid sequences of bHLH proteins.

The coding domain sequences (CDS) and DNA sequences of tomato *bHLH* genes were used to assess gene structure using GSDS (http://gsds.cbi.pku.edu.cn/) [42].

### Collinear correlations of bHLH genes in the tomato, potato, and *Arabidopsis* genomes

OrthoMCL program (http://www.orthomcl.org/cgi-bin/OrthoMclWeb.cgi) [43] was used to identify the orthologous and paralogous genes in tomatoes, potatoes and *Arabidopsis*. Briefly, the tools BLASTP, with an e-value ≤ 1e^−10^, and orthomclPairs were used to find orthologs, inparalogs and coorthologs in these three species. The Circos tool was used to link these genes to chromosomes [44]. In addition, the relationships of orthologous and paralogous genes in these three species were also shown using the Circos tool [44]. The bHLH genes in tomato were searched for duplication events (e value <1e^−10^, identity >90%).

### Chromosome distribution and gene duplications

To determine the physical locations of *bHLH* genes, the starting and ending positions of all *bHLH* genes on each chromosome were obtained from the tomato database. The MapInspect software was used to draw the images of the locations of the tomato *bHLH* genes (http://mapinspect.software.informer.com/). We used the plant genome duplication database (PGDD, available at http://chibba.agtec.uga.edu/duplication/) to retrieve the duplicate chromosomal blocks and then identify the *bHLH* genes in the duplication block which allowed us to identify duplicate tomato *bHLH* genes [45]. The PGDD is a public database used to identify and catalogue plant genes in terms of intra-genomic or cross-genomic syntenic relationships.

### RNA data collection and data mining

Transcriptomic data of TYLCV-resistant breeding line, CLN2777A (R) and susceptible breeding line, TMXA48-4-0 (S) with uninfected (0 dpi) and mixed infection samples of 3, 5, and 7 days post infection (357 dpi) were downloaded from NCBI SRA database (SRA097118) and analyzed as described in a previous study [33]. Enrichment of gene ontology (GO) categories was performed with an agriGO analysis toolkit (http://bioinfo.cau.edu.cn/agriGO/) [46] using the TopGO ‘elim’ algorithm [47] for the aspects ‘biological process’ and ‘subcellular localization’. The selected categories were sorted from the lowest to the highest P value (*P* < 0.01).

### Validation of differentially expressed genes by quantitative RT-PCR

Four bHLH genes with differentially expressed in R or S lines were selected and subjected to quantitative RT-PCR validation (Additional file 1: Table S1). Primers for quantitative RT-PCR were designed using Primer5 software and primer specificity was evaluated by blasting primer sequences against the NCBI database. PCR amplifications were performed in a real-time thermal cycler qTOWER 2.0/2.2 (Analytik Jena, Germany) with 15 μl of final volumes containing 1.0 μl of cDNA, 0.5 μl each primer (10 μM), 6 μl of sterile water, and 7.5 μl (2×) SYBR Premix *ExTaq*™ II Kit (TaKaRa, Japan). The conditions for amplification were as follows: 5 min of denaturation at 95°C followed by 40 cycles of 95°C for 10 s, 60°C for 20 s, and 72°C for 10 s. The expression levels of selected genes were normalized to *α-Tubulin* (Solyc04g077020.2) expression [33]. Relative gene expression was calculated using the 2^-ΔΔCT^ method [48]. Three biological replicates were performed for each of the selected genes.

### Validation of candidate genes with virus-induced gene silencing (VIGS) and cell death analysis

The tobacco rattle virus (TRV) mediated VIGS system was used to silence a *bHLH* gene (Solyc10g008270.2) [49]. Briefly, pTRV-containing *Agrobacterium* EHA105 was cultured in liquid LB medium and resuspended in infiltration medium at an O.D. value of 2.0 and cultured at room temperature for 4 h. Three week old seedlings were infiltrated by pressure inoculation in the leaves with a needleless syringe. For the VIGS experiments, agro infiltration was performed two weeks after TRV inoculation.

Death cells were identified by staining with lacto-phenol trypan blue and DAB as previous described [50,51]. To visualize cell death, stem were stained by boiling in lacto-phenol trypan blue (10 ml lactic acid, 10 ml glycerol, 10 g phenol, and 10 mg trypan blue, dissolved in 10 ml distilled water), followed by destaining with chloral hydrate (2.5 g ml^−1^). Then the death cell was examined with Ni-U microscope (Nikon, Japan).

## Results

### Identification and classification of bHLH proteins in tomato

To identify the putative bHLH proteins in the tomato genome, a Hidden Markov Model search resulted in the identification of 152 bHLH proteins (Additional file 1: Table S2). To verify the reliability of our criteria, we performed simple modular architecture research tool (SMART) analysis of 152 putative SlybHLH protein sequences and found all of them had a typical bHLH domain. The number of bHLH TFs in tomatoes exceeded that of many metazoans and fungi, but was less than that found in some plants, such as rice (170), Chinese cabbage (230), potatoes (127), soybeans (289) and maize (289) [18]. In addition, the density of bHLH proteins in the entire tomato (0.198) and potato (0.175) genomes was found to be less than that in most plant species, such as *Arabidopsis* (1.111) and rice (0.46) [18]. Most Angiosperm plant lineages have experienced one of more rounds of ancient polyploidy [45]. And the genomes of tomato and potato have undergone recent triplication events, whereas few individual tomato/potato genes remain triplicated [21]. Therefore, this event might lead to relatively fewer bHLH genes in tomato compared to other plant species.

### Multiple sequence alignments, predicted DNA-binding ability and conserved residues

**Figure 1:**
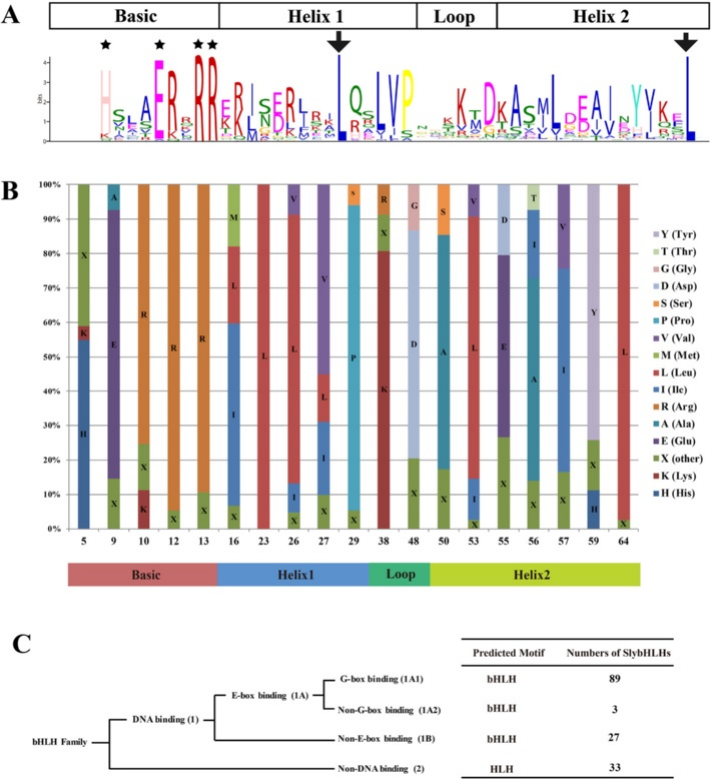
**The characterization and distribution of the bHLH domains. A**. Sequence logo of the SlybHLH domain by MEME. The H5, E9 and R12 amino acids in the basic domain that are important for DNA binding are indicated by stars. Amino acids important for dimerization of the helix-loop-helix domain are indicated by arrows. **B**. Distribution of amino acids in the bHLH consensus motif among tomatoes. The numbers of horizontal ordinate refer to the positions of the residues in the alignments of the studies. **C**. Predicted DNA-binding characteristics of the bHLH domain of SlybHLH proteins.

To examine sequence features of these tomato bHLH domains, multiple sequence alignment of the 152 bHLH amino acid sequences were performed. There were four conserved regions in the bHLH domain sequences, including one basic region, two helix regions and one loop region (Figure 1A, Additional file 1: Table S3). The basic regions have five basic residues, but five of these proteins did not have the basic region (Additional file 2: Figure S1). The loop was found to be the most divergent region in terms of both length and amino acid composition. From the alignment, 19 residues were identified that were identical in at least 50% of the 152 tomato bHLH domains (Figure 1B). Among these 19 residues, nine residues were present in more than 75% sequences (Glu-9, Arg-10, Arg-12, Arg-13, Leu-23, Leu-26, Lys-38, Leu-53 and Leu-64 in this alignment).

Five residues (His-5, Glu-9, Arg-10, Arg-12, and Arg-13), five residues (Ile-16, Leu-23, Leu-26, Val-27, and Pro-29), two residues (Lys-38 and Asp-40) and seven residues (Ala-50, Lys-53, Glu-55, Ala-56, Ile-57, Tyr-59, and Lys-64) made up the basic region, the first helix region, the loop region and the second helix region, respectively. All of these conserved residues were consistent with previous studies [3,17,18]. The Leu-23 in the basic region was conserved in all 152 bHLH proteins, suggesting that this residue is extremely important for promoting the formation of dimerization among bHLH proteins [5].

The basic region of the bHLH domain can bind to DNA and is critical for function [52]. Using the criteria described by Massari and Murre, the SlybHLH proteins were divided into several categories based on sequence information in the N-terminal region of the bHLH domains (Figure 1C, Additional file 1: Table S2) [52]. As was done with *Arabidopsis* and Chinese cabbage, the SlybHLH proteins of tomato were also divided into two major groups according to 17 N-terminal amino acids within bHLH protein domain, including 119 DNA-binding and 33 non-DNA binding proteins. The DNA-binding bHLHs were further divided into two groups with different predicted target sequences depending on the presence or absence of residues Glu-9 and Arg-12 in the basic region. Group (1A) proteins had 92 putative E-box-binding proteins with conserved Glu-9/Arg-12 residues and Group (1B) proteins had 27 non-E-box-binding proteins lacking these residues (Figure 1C). The three residues in the basic region of the bHLH domain, His/Lys-5, Glu-9 and Arg-1, were found to constitute the classic G-box-binding region [52]. Group (1A) can therefore be subdivided further into two subgroups: 1A1, whose 89 proteins are predicted to bind G-boxes, and 1A2, whose three members are predicted to bind other types of E boxes (non-G-box proteins).

### Phylogenetic analysis of the bHLH transcription factor family

**Figure 2:**
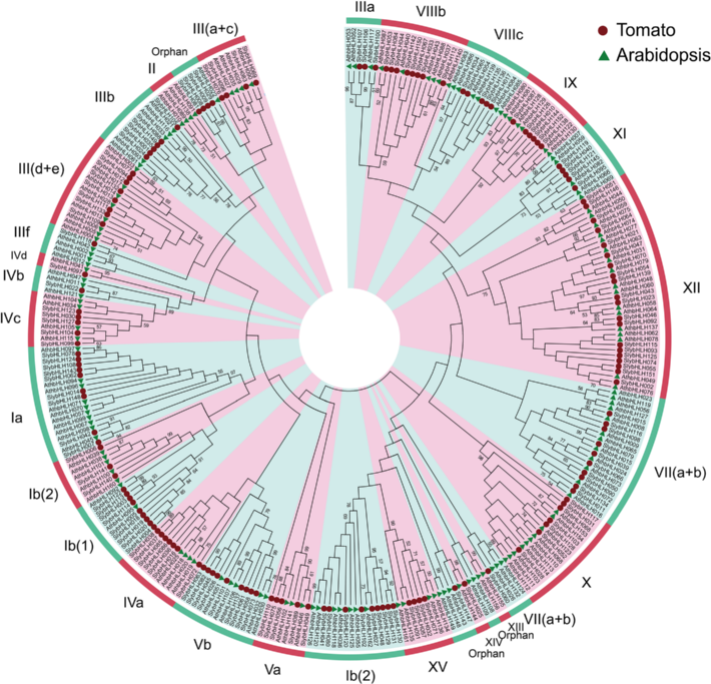
**Phylogenetic tree constructed from the neighbor-joining method using the bHLH transcription factor domain in tomato.** The phylogenetic tree was constructed using MEGA6 software. The numbers are bootstrap values based on 1000 iterations. Only bootstrap values larger than 50% support are indicated.

To assess the evolutionary relationships of the *SlybHLH* genes, an NJ phylogenetic tree was generated using the multiple sequences alignments of the conserved bHLH TF domains in tomato and *Arabidopsis* with a bootstrap value of 1,000. Twenty-six subfamilies were identified according to the clades support values, topology of the tree, and classification of the *Arabidopsis* [3,19]. No SlybHLH proteins in the XIII, II, subfamilies relative to those of *Arabidopsis*, therefore, tomato contained 24 bHLH subfamilies in our analysis (Figure 2). To further validate the reliability of the NJ tree with MEGA 6.0, NJ and maximum parsimony analysis was also used to generate phylogenetic trees using PHYLIP software (Additional file 3: Figure S2 and Additional file 4: Figure S3). 96.7% (147/152) of the SlybHLH proteins with NJ model using PHYLIP software were placed into the same subfamilies as those in the NJ tree with MEGA 6.0, indicating that both methods are in very good agreement.

In order to assess differences in protein structure, MEME was used to identify conserved motifs in the tomato bHLH proteins. Ten conserved motifs were identified and named motif 1 through motif 10. In general, the bHLH proteins were clustered in the same subfamilies and shared similar motif compositions, which indicated functional similarities among members of the same subfamilies [3]. The tomato bHLH proteins were found to have a similar structure for every subfamily (Additional file 4: Figure S3). The pattern of intron position can also provide important evidence to support phylogenetic relationships in a gene family. Here, GSDS tools were used to show the gene structures for *SlybHLH* genes (Additional file 5: Figure S4). Among the 152 tomato *bHLH* genes, the number of introns ranged from 0 to 10, and most members of the same subfamilies had similar intron/exon structures. For example, the members of subfamilies III(d + e) and XV each have only one intron. These results demonstrated that proteins within the same subfamily share close evolutionary relationships.

### Collinear correlations of bHLH genes in tomatoes, potatoes, and *Arabidopsis*

**Figure 3:**
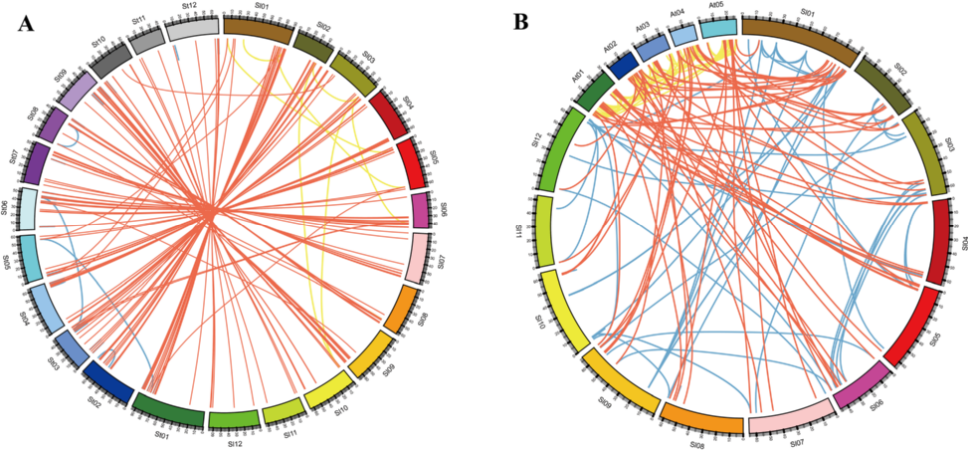
**Comparative analysis of synteny and expansion of bHLH genes. A.** Twelve tomato chromosomes (Sl01-Sl12) and 12 of potato chromosomes (St01-St12) maps were based on the orthologous and paralogous pair positions and demonstrate highly conserved synteny. The red lines represent the orthologous bHLH genes between tomato and potato. The blue and yellow lines represent the paralogous bHLH genes in potato and tomato, respectively. **B**. Twelve tomato (Sl01-Sl12) and Arabidopsis chromosome (A01-A05) maps were based on the orthologous and paralogous pair positions and demonstrate highly conserved synteny. The red lines represent the orthologous bHLH genes between tomato and *Arabidopsis*. The blue and yellow lines represent the paralogous bHLH genes in tomato and *Arabidopsis*, respectively.

The *Solanum* lineage has experienced two consecutive genome triplications: one is ancient and shared with rosids, and the other more recent [21]. In this study, the correlation between tomato, potato, and *Arabidopsis bHLH* genes was analyzed using the OrthoMCL program. Here, 167 gene pairs were found to be orthologous between tomatoes and potatoes, but only 61 orthologous pairs were found between tomato and *Arabidopsis* (Additional file 1: Table S4). These results were consistent with the close relationship of tomatoes and potatoes. Among the orthologous gene pairs shared by tomatoes and potatoes, each tomato *bHLH* gene had one to four potato *bHLH* genes. These results demonstrated that *bHLH* TF genes in potato were duplicated accompanied with evolution processes. In addition, paralogous *bHLH* gene pairs were also analyzed. A total of 61, 72, and 81 bHLH gene pairs were identified in *Arabidopsis*, tomatoes, and potatoes, respectively (Additional file 1: Table S5). Visualization of the relationships of paralogous and paralogous *bHLH* genes among these three species was performed using the Circos software (Figure 3).

### Chromosome distribution and gene duplication of the bHLH TF family

**Figure 4:**
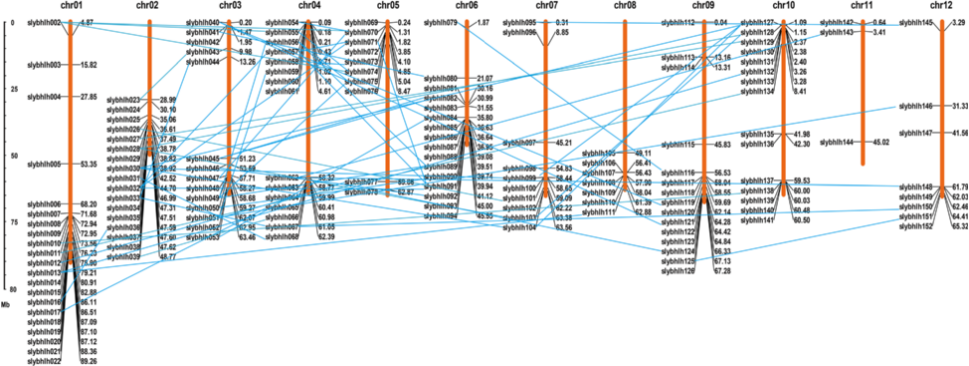
**Distribution of 152 bHLH genes on the twelve tomato chromosomes using MapInspect software.** Only SlybHLH001 could not be anchored onto a specific chromosome. The scale is in megabases (Mb). The duplicate bHLH genes are connected with blue lines.

The physical map positions of the *bHLH* genes on tomato chromosomes were identified (Figure 4). Among the 152 *bHLH* TFs, 151 were mapped onto the twelve tomato chromosomes except *SlybHLH001*. Most *bHLH* TFs were found on chromosome 01 (21, 13.8%) and 02 (18, 11.1%). In contrast, there are only 3 (2.0%) and 7 (4.6%) *bHLH* TF genes on chromosome 11 and 08, respectively. Furthermore, *bHLH* genes were found to be mapped on the chromosomes with an obviously uneven distribution, and some *bHLH* genes gathered on part of the chromosome. Relative high densities of bHLH genes were observed in some chromosomal regions, including the bottom of chromosomes 01, 02, 06, and 09. For example, 17 genes clustered in the end of chromosome 01 and 02 with density of 0.8 and 0.86 genes per Mb, respectively. In contrast, several large chromosomal regions lacked bHLH genes, such as the top half of chromosomes 02 and 08 and the central section of chromosomes 03, 04, 05, and 11 (Figure 4).

Previous reports have analyzed duplication events in rice and Chinese cabbage [17,18]. In the current analysis, we first retrieved the genome chromosome blocks in the tomato with PGDD database and identified the 382 duplicate blocks. A total of 59 duplicate bHLH genes pairs were located in these blocks (Figure 4). These duplication bHLH genes are derived from the same subfamily, indicating that members of some bHLH subfamilies originated from the duplication events.

### Differential expression of *SlybHLH* genes in response to TYLCV infection

**Figure 5:**
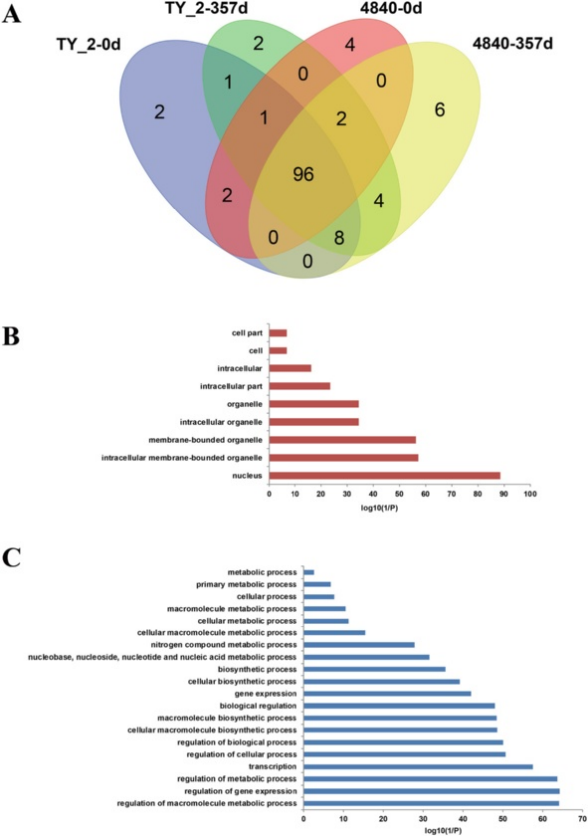
**The expression levels and related functions of bHLH genes. A**. Venn’s diagram of the cross comparison of expressed genes based on RNA-seq data. **B**. Gene ontology (GO) enrichment analysis of expressed bHLH genes involved in the biological processes. **C**. Subcellular location of bHLH genes.

The RNA-seq technology has been shown to provide precise digital information related on gene expression and can discriminate genes of high sequence identity [53]. Using this technology, global gene expression changes of the leaves of two tomato breeding lines, TYLCV-resistant CLN2777A and TYLCV-susceptible TMXA48-4-0, have been analyzed before (0 dpi) and after (357 dpi) TYLCV infection with viruliferous whiteflies [33]. A total of 34,831 transcripts were detected from R and S lines by alignment to the tomato genome including the 1,386 novel transcripts predicted in tomatoes. The expression levels of mapped genes were normalized with a value of fragments per kilobase of exon per million fragments mapped (FPKM). If the FPKM value of gene was above zero, the gene was considered expressed. On the basis of this criterion, the expression level of 152 *SlybHLH* genes was confirmed in the S and R line with 0 and 357 dpi. A subset of 96 *SlybHLH* genes (63.2%) was expressed under both conditions (Figure 5A). GO enrichment analysis revealed that the products of most of *SlybHLH* genes were localized in the nucleus (Figure 5B). In addition, some biological processes such as ‘regulation of macromolecule metabolic process’ (GO: 0060255), ‘regulation of gene expression’ (GO: 0010468) and ‘regulation of metabolic process’ (GO: 0019222) were overrepresented among all *SlybHLH* genes, indicating that the *bHLH* genes were involved in transcription and metabolic regulation (Figure 5C).

**Figure 6:**
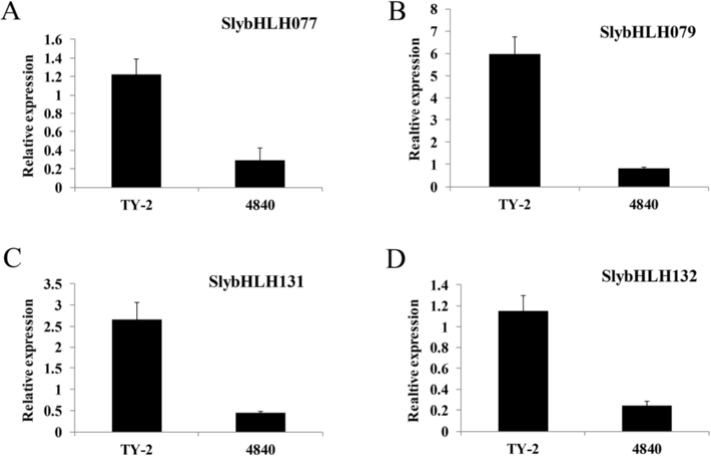
**Quantitative RT-PCR was used to measure the relative expression levels of five pathogen resistance related genes in the R (TY-2) and S (4840) lines, with tomato α-Tubulin (Solyc04g077020.2) as an internal reference. A**. SlybHLH077. **B**. SlybHLH079. **C**. SlybHLH131. **D**. SlybHLH132.

Out of 152 *SlybHLH* genes in the current study, only four were differentially expressed before and after TYLCV infection (log2 fold change >1 and false discovery rate < 0.05) (Table 1). Among the four differentially expressed *SlybHLH* genes, *SlybHLH131* (*Solyc10g008270.2.1*) was up-regulated in the R line and down-regulated in the S line after TYLCV infection. The expression level of four differentially expressed genes was determined using quantitative RT-PCR to validate the gene expression data from RNA-seq (Additional file 1: Table S1 lists the primers). The results demonstrated that all tested genes revealed a similar trend of transcript accumulation as in RNA-seq analysis (Figure 6).

**Table 1:** **Differentially expressed** ***SlybHLH*** **genes between before and after TYLCV infection**

	**TY-2**			**4840**		
	**0 dpi**	**357 dpi**	**Fold change**	**0 dpi**	**357 dpi**	**Fold change**
SlybHLH079	1.874	30.704	4.034	9.571	6.706	−0.513
SlybHLH131	0.673	18.369	4.770	26.498	1.373	−4.270
SlybHLH077	3.280	3.245	−0.015	39.521	2.812	−3.813
SlybHLH132	1.761	3.506	0.993	28.111	1.506	−4.222

### VIGS validation of *SlybHLH131* gene

**Figure 7:**
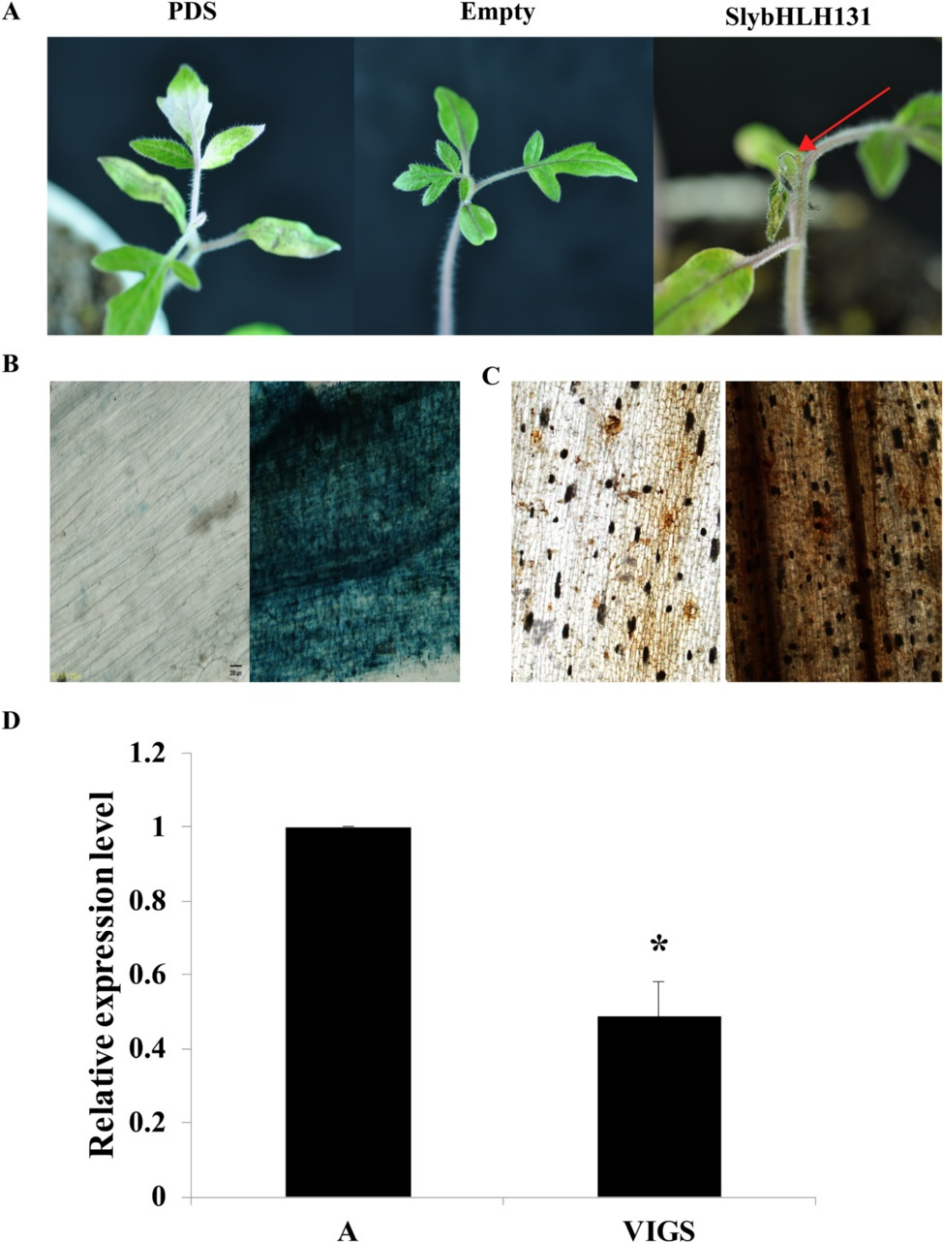
**Validation of the bHLH gene (** ***SlybHLH131*** **) with virus-induced gene silencing. A**. Cotyledon agroinfiltration of TRV vectors was carried out in the S plantlets at the cotyledon stage. R plants treated with Phytoene desaturase (PDS) gene silencing constructs pTRV1 and pTRV2 - (PDS) showed bleached areas in leaflets (left). S plants treated with pTRV1 and pTRV2 vectors showed the normal phenotype (middle). S plantlets treated with *SlybHLH131* gene silencing constructs pTRV1 and pTRV2 - (*SlybHLH131*) showed a cell death phenotype (right). Arrow indicates cell death. **B**. Trypan blue staining of VIGS and empty vector lines leaves. **C**. DAB staining of VIGS and empty vector lines leaves. **D**. Relative expression of *SlybHLH131* transcript using real-time RT-PCR analysis in the VIGS-treated lines 20 days after agroinfiltration with TRV vectors. Tomato α-Tubulin (Solyc04g077020.2) was used as an internal reference. Error bars represented SE of three biological replicates and asterisks indicate significant differences by Student’s *t* test for *P* < 0.05.

To investigate the role of TYLCV as it related to resistant in tomato, the *SlybHLH131* gene was further challenged with TYLCV after VIGS at the cotyledon stage. One month after agroinfiltration, the success of the TRV silencing system was confirmed by the appearance of cell death in the leaves of S plantlets treated with pTRV1 and pTRV2- *SlybHLH131* (Figure 7A). We also observed the cell death development under Ni-U microscope and found that the VIGS lines with *SlybHLH131* gene triggered a rapid cell death response in comparison with empty vector lines by trypan blue and DAB staining (Figure 7B and C). A quantitative RT-PCR analysis showed there were significantly fewer *SlybHLH131* transcripts in *SlybHLH131*-silenced tomato leaves during TRV infection (Figure 7 D), indicating that *SlybHLH131* was effectively silenced in tomatoes.

## Discussion

The Solanaceae is one of the largest angiosperm genera and includes many different vegetables consumed by humans. In recent years, many plants in this genera have been sequenced, including tomatoes [21], potatoes [54], peppers [55], and tobacco [56]. With the rapid development in bioinformatic analyses, the information stored in various genomes may be explored to elucidate the mechanisms that regulate the development and response to biotic and abiotic stresses. Tomatoes are major crop plant and a model system for fruit development. As of 2011, tomato production had doubled from 24 million tons in 2001 [33]. However, tomato yellow leaf curl virus disease causes huge losses in tomato production worldwide. This disease is caused by different related be gomovirus species. A previous study extended our basci understanding of the response of tomato to TYLCV infection by comparing whole transcriptome expression changes between a TYLCV-resistant line and a TYLCV-susceptible line. In present study, 152 bHLH transcription factor genes were identified in tomatoes and their differential expression was analyzed in R and S lines before and after TYLCV infection. Four differentially expressed genes were identified in R and S lines, in which *SlybHLH077* and *SlybHLH079* were derived from the chromosome 5 and 6, and *SlybHLH131* and *SlybHLH132* were mapped on the chromosome 10. These genes are not located in the region of five known loci (*Ty-1* to *Ty-5*), so they are not the most important genes but might be involved in the regulation network of TYLCV resistance. Phylogenetic analysis of the bHLH domain allows division of the SlybHLH family into 23 subfamilies. The clustering of the members within these subfamilies was further supported by additional analysis with regard to other criteria, such as predicted DNA binding capacity and sequence specificity, exon/intron distribution pattern with the domain. These data support the general conclusion that members within subfamilies may have recent common evolutionary origins resulting from various genomic duplication events. They may have related molecular functions. The bHLH subfamily IIId transcription (bHLH3, bHLH13, bHLH14, and bHLH17) function function redundantly to negatively regulate jasmonate (JA) responses in *Arabidopsis* [57]. However, the strong sequence diversity outside of the bHLH domain across the members of the SlybHLH family suggests that the expansion of this family in tomatoes involved extensive domain shuffling, as in other organisms. However, the non-bHLH amino acid motifs are conserved in each of the bHLH subfamilies (Additional file 5: Figure S4), suggesting that the conservation of these extra domains during plant evolution may have been essential to the function of the bHLH proteins in their respective subfamilies [58].

The core DNA binding domain of the bHLH proteins contained the basic region of the bHLH domain and these residues were found to recognize and bind to the core hexanucleotide [52]. The amino acid sequence in this region provides the major subdivision of the bHLH family, dividing these proteins into those that are predicted to bind DNA and those that are not (Figure 3). Key residues in this region confer the capacity to discriminate the variants of the hexanucleotide motif with the canonical E-box (CANNTG) and non-E-box motifs. Additional residues within the basic region confer further DNA binding site sequence selectivity. These include G-box and non-G-box core motifs. The current analysis showed there are 92 E-box binding bHLH proteins in tomatoes, and this ration was lower than in *Arabidopsis* and Chinese cabbage, indicating that many different binding motifs exist in the tomato bHLH protein family.

Most bHLH proteins identified so far were functionally characterized in *Arabidopsis*, and their roles include plant development, fruit dehiscence, phytochrome signaling, hormone signaling and stress responses, such as cold, heat, abscisic acid, jasmonic acid, and the light signaling pathway [20]. Only two bHLH genes have been functionally characterized in tomato. One is the *FER* gene (SlybHLH083), which is involved in the response to iron acquisition and supply [34]. Another is *Style2.1* (SlybHLH031), that is associated with the evolution of self-pollination; its natural genetic variation in the promoter is responsible for evolution from allogamy to autogamy in the cultivated tomatoes [35]. In the current RNA-seq data set, the *FER* gene was not expressed in any of the four samples, and the *Style2.1* gene was expressed but not differentially expressed in R or S lines after TYLCV infection.

Members of the same plant bHLH subfamily are frequently involved in the same biological processes. Usually the functions of these proteins overlap, causing them to be partially or totally redundant [19]. The characterized function of bHLH in other species can help the user to predict the function of tomato bHLH in the same subfamily. Jasmonates (JA) are lipid-derived hormones that regulate plant responses to stresses such as wounding and pathogens invasion [59]. JA negatively regulates plant growth and is considered to modulate the distribution of energy to defense responses [60]. In *Arabidopsis*, the bHLH subfamily IIId members (bHLH3, bHLH13, bHLH14, and bHLH17) act as transcription repressors and function redundantly to negatively regulate JA response. In tomatoes, eight members of the III(d + e) subfamily have been identified, so these bHLH TFs might be involved in the JA response network and defense against TYLCV infection. *SlybHLH131*, a member of the Ib(2) subfamily was up-regulated in the R line, and down-regulated in the S line. In barley and rice, this subfamily is involved in Fe uptake and homeostasis [61,62]. This suggests that *SlybHLH131* might perform some previously unknown function in TYLCV infection. Therefore, our results will pave the way for studies of new functions of bHLH genes in TYLCV infection and will further our understanding of this gene family under other biotic and abiotic stresses in tomato.

## Conclusion

An extensive analysis of the tomato bHLH genes was performed, identifying 152 bHLH TFs in the entire tomato genome. These genes can be divided into 24 subfamilies using phylogeny, protein motifs, and gene structures. This phylogenetic analysis is in consistent with previous results. The members of subfamilies may share conserved functions not shared by other species. The pattern of expression of *SlybHLH* genes was observed in R and S lines infected with TYLCV. Results showed that *SlybHLH131* might be involved in the TYLCV infection by VIGS. In summary, this is the first comprehensive and systemic analysis of bHLH transcription factors in tomatoes, and the results of this study revealed the importance of bHLH genes during TYLCV infection. They may also provide new opportunities for the investigation of previously unknown mechanisms by which tomatoes tolerate TYLCV infection. Furthermore, our results have established a solid foundation for future studies of other biotic and abiotic stresses using biochemical and physiological approaches that will probably reveal the functional significance of this family in tomato.

## Additional files

Additional file 1: Table S1. The primer sequences used for quantitative real-time PCR amplification of actin and SlybHLH131. **Table S2.** Summary of bHLH genes in tomato genome. **Table S3.** The bHLH domain sequences of tomato. **Table S4.** The orthologous bHLH genes in tomato, potato and Arabidopsis genome. **Table S5.** The paralogous bHLH genes in tomato, potato and Arabidopsis genome. **Table S6.** The expression of 152 SlybHLH genes in TY-2 and 4840 after TYLCV infection. Additional file 2: Figure S1. Alignment of all the bHLH domain of tomato proteins. Shown at the top are the boundaries used in this study to distinguish the DNA-binding basic region, the two a-helices and the variable loop region. Additional file 3: Figure S2. The NJ phylogenetic tree of bHLH proteins motif using PHYLIP software. Additional file 4: Figure S3. The NJ phylogenetic tree and conserved motif compositions of tomato. The neighbor-joining tree of tomato bHLH genes and their motif locations. Additional file 5: Figure S4. The NJ phylogenetic tree and SlybHLH gene structure of tomato. The yellow and green blocks indicate introns and exons, respectively.
