# Supplementary figures and images for: Genome-wide analysis of bHLH transcription factor and involvement in the infection by yellow leaf curl virus in tomato (Solanum lycopersicum)
Source: BMC Genomics. 2015 Feb 5;16(1):39. doi: 10.1186/s12864-015-1249-2 (PMC4333901; doi:10.1186/s12864-015-1249-2)

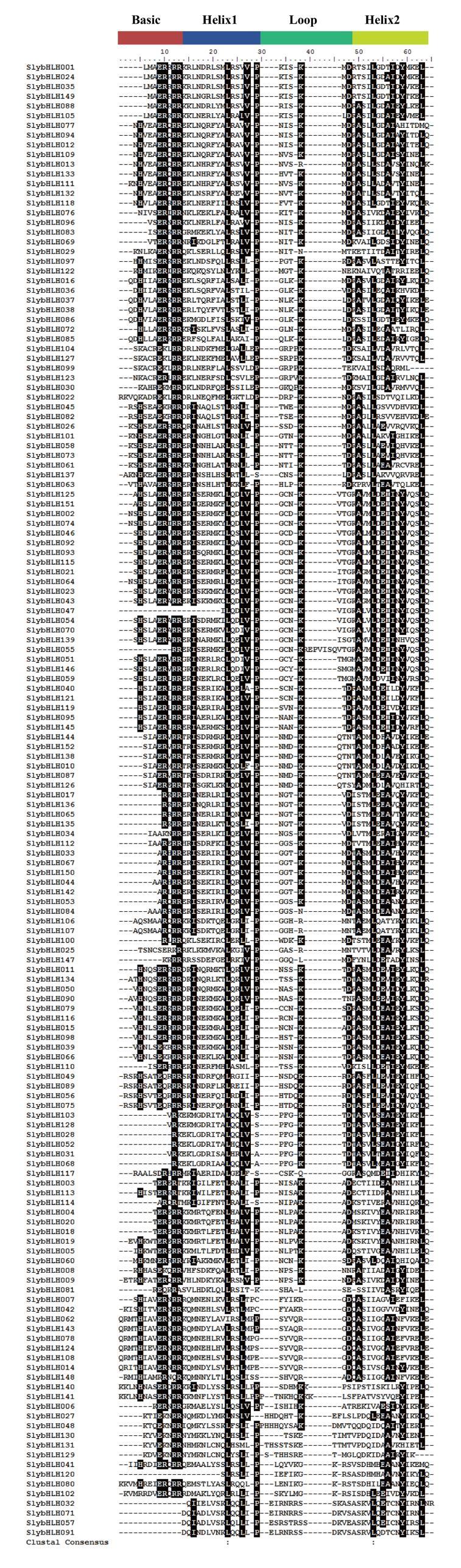

Supplement: Additional file 2: Figure S1. — Alignment of all the bHLH domain of tomato proteins. Shown at the top are the boundaries used in this study to distinguish the DNA-binding basic region, the two a-helices and the variable loop region. [file 12864_2015_1249_MOESM2_ESM.jpeg]

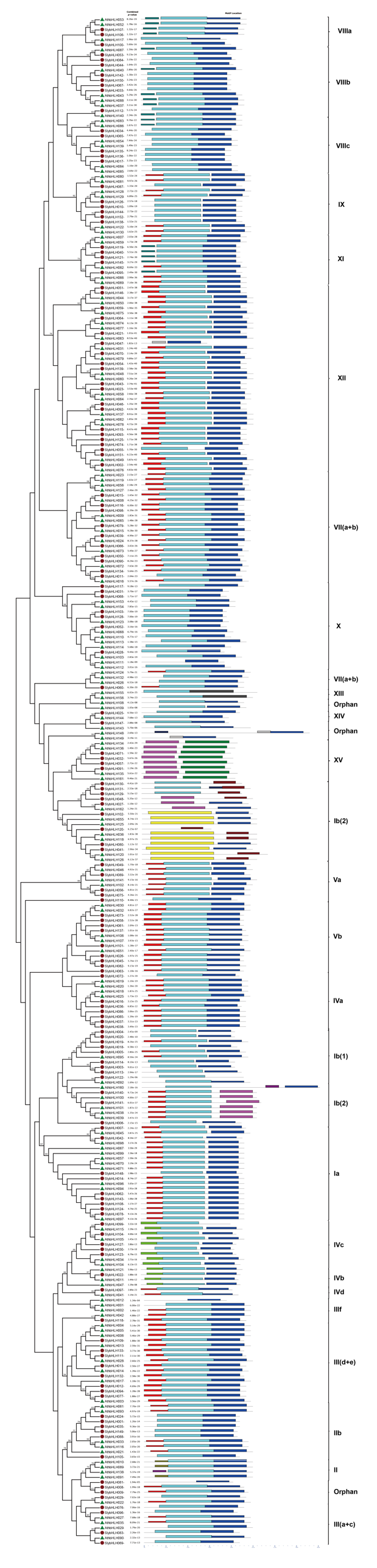

Supplement: Additional file 4: Figure S3. — The NJ phylogenetic tree and conserved motif compositions of tomato. The neighbor-joining tree of tomato bHLH genes and their motif locations. [file 12864_2015_1249_MOESM4_ESM.jpeg]

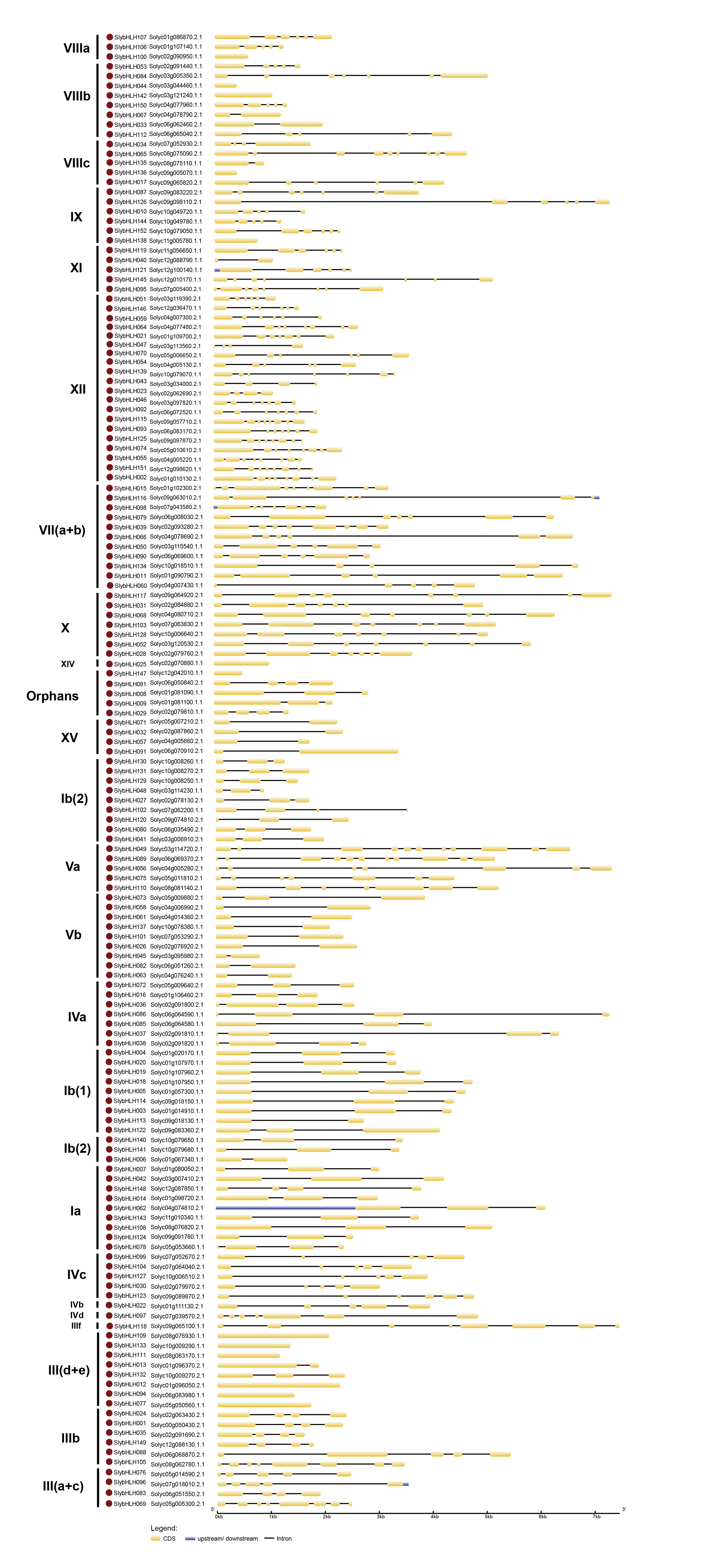

Supplement: Additional file 5: Figure S4. — The NJ phylogenetic tree and SlybHLH gene structure of tomato. The yellow and green blocks indicate introns and exons, respectively. [file 12864_2015_1249_MOESM5_ESM.jpeg]
